# Preparation and Application of Responsive Nanocellulose Composites

**DOI:** 10.3390/polym16111446

**Published:** 2024-05-21

**Authors:** Yanhui Zhou, Lu Zhang, Yuan Li

**Affiliations:** 1 School of Applied Foreign Languages, Guangdong Industry Polytechnic, Guangzhou 510300, China; 2003106089@gdip.edu.cn; 2 Guangdong Engineering Technology Research Center of Biomaterials, Institute of Biological and Medical Engineering, Guangdong Academy of Sciences, Guangzhou 510316, China; zhangluend@163.com

**Keywords:** semi-IPN, cellulose nanofibrils, responsive, dye removal

## Abstract

Cellulose nanofibrils/poly(N-Isopropylacrylamide) semi-interpenetrating networks (MMCNF-PNAs) were synthesized using an in situ fabrication (semi-IPN). The polymerization of N-isopropylacrylamide (NIPAM) (free radical) was conducted in the presence of magnetic modified cellulose nanofibrils (MMCNFs). The adsorption behaviors and surface morphology of the synthesized adsorbents were investigated systematically. The adsorption behaviors of the as-prepared MMCNF-PNA towards methylene blue (MB, as the model contaminant) dye was studied, and the optimal adsorption conditions were also studied. The adsorption processes could be well fitted using pseudo-second-order and Elovich kinetic models. Meanwhile, Langmuir and Freundlich isotherm models were used to fit the adsorption which occurred at 25, 37 and 65 °C. The corresponding results showed that the Freundlich isotherm model fitted the adsorption process better, indicating that the dye’s adsorption happened via heterogeneous adsorptive energies on the prepared MMCNF-PNAs. Their desorption and reusability were also studied to verify magnetic responsivity. To sum up, MMCNF-PNAs are promising magnetic and thermal stimuli-responsive adsorbents, showing a controlled adsorption/desorption process.

## 1. Introduction

The presence of dyes in wastewater has attracted the increasing concern due to the growing amount of industrialization in the world. Dyes can enter water through the disposal of wastes from the textile, printing and dyeing industries. The dyes from different industries include methylene blue (MB), methyl violet (MV), methyl orange (MO), methyl red (MR), peacock green (PG), etc. High concentrations of these dyes are dangerous to the health of humans and therefore need to be removed from wastewater. Adsorption is becoming an increasingly common method of dye removal from wastewaters. Adsorption with activated carbon has been found to be an effective method of removing dyes from water without the production of any harmful by-products [1]. However, even though activated carbon is a widely used adsorbent, it still has relatively high costs associated with it [2]. In this case, the use of low-cost adsorbents, such as modified cellulose materials, has gained much interest for the removal of heavy metal ions from wastewater.

The use of cellulose and its derivatives (nanocellulose or micro-fibrillated cellulose) as an adsorbent is becoming increasingly common, as cellulose is the most abundant and renewable polymer resource in the world. This is beneficial, as a disadvantage to using adsorption as a method to remove dyes from wastewater is the high cost of adsorbents. However, selectivity and the adsorption capacity are limited when unmodified cellulose is used as an adsorbent directly. In addition, the hydroxyl groups on the chains of cellulose can react easily, introducing new functional groups such as -NH_2_, -COO-, -COOH, or -SO_3_H groups via various modification methods including etherification, halogenation, esterification, grafting and oxidation [3]. The mentioned approaches can change the properties of cellulose, including hydrophobicity or hydrophilicity, adsorptive capability and elasticity [4]. Renewable cellulose nanocrystals (CNCs) and cellulose nanofibrils (CNFs) have been seriously studied in recent years. CNCs are usually isolated using strong acid hydrolysis. The mechanical fabrication methods of CNFs include high-pressure homogenization, refining and grinding [5]. Such approaches utilize fewer chemicals compared to those methods of preparing CNCs. CNFs combine the advantages of nanomaterials and bio-based materials, which makes them an ideal absorbent for introducing the active functional groups.

Recently, grafting modification has been suggested as a convenient approach to introducing new chemical and physical properties to cellulose [6]. The most widely reported responsive polymers are thermal-responsive poly (N-isopropyl acrylamide) (PNIPAM) and pH responsive polymers with carboxyl groups (polyacrylic acid (PAA)). In recent years, materials with PNIPAM and PAA have been widely used in medicine and biotechnology [7]. Furthermore, the graft modification of cellulose with PNIPAM is reported to be a novel method of cellulose modification. The responsive polymers can be used to modify the cellulose materials using grafting, which is known as responsive modification of cellulose. Cha et al. [8] synthesized NIPAM-based responsive hydrogels using CNCC (carboxylated nanocrystalline cellulose) as the raw material. The as-prepared hydrogel showed thermal/pH sensitivity. Soyeon and Kevin [9] reported on hydrogel prepared using NIPAM and AA as the raw materials (poly (NIPAAm-co-AAc)). The corresponding hydrogel was proved to create environmentally responsive artificial extracellular matrixes. From the methods mentioned above, there is great potential for cellulose-based materials to be applied as ideal candidates to prepare responsive or smart bio-adsorbents. In addition, some functional fillers such as graphene oxide and Fe_3_O_4_ can be introduced as reinforcing agents to enhance the properties of composites for various applications. Dai et al. developed a pH/magnetic responsive hydrogel consisting of pineapple peel carboxymethyl cellulose (PCMC), regenerated nanocellulose (rPPNc) and polyvinyl alcohol (PVA), accompanied by in situ incorporation of Fe_3_O_4_. The resulting hydrogels can be seen as promising candidate materials for drug delivery systems due to their characteristics of biocompatibility, pH and magnetic sensitivity [10].

An interpenetrating network (IPN) prepared from polymers is obtained when a network of two or more polymers are interlaced with each other. However, a semi-interpenetrating network (semi-IPN) is created when one of the polymers is not fully cross-linked. In this work, we developed a magnetically modified cellulose nanofibril and thermal-responsive semi-interpenetrating network (semi-IPN) adsorbent as a green-based candidate for dye removal in aqueous solutions. Generally, the surface morphology can be modified by swelling behaviors with temperature changes. As a result, the adsorption/desorption process can be changed effectively by varying temperature. The magnetic modification was carried out using in situ incorporation of Fe_3_O_4_, followed by the responsive modification using a free radical copolymerization. Two cationic dyes, methylene blue (MB) and methyl violet (MV), were used as the target contaminants. The proper conditions for the preparation processes were studied, followed by investigation of the morphology characterization, the adsorption and desorption mechanism. The preparation method of this work consisted of the magnification of cellulose and free radical polymerization using thermal-responsive monomer as the modifier. The method could also be applied for the modification of other cellulose materials, and it is suitable for industrial applications.

## 2. Materials and Methods

### 2.1. Materials and Reagents

Cellulose nanofibrils (CNF, diameter 4–10 nm; length 100–500 nm; solid content 4.5%) were purchased from Guilin Qihong Technology Co., Ltd., Guilin, China; this product was prepared using a mechanical method. Wood cellulose was used as the ray material. Potassium persulfate (KPS), N-isopropylacrylamide (NIPAM), acrylic acid (AA), N-N-Methylenebisacrylamide (MBA), methylene blue (MB) and methyl violet (MV), FeCl_3_·6H_2_O, FeCl_2_·4H_2_O, ethanol, acetone, ammonia and other reagents were used as purchased and received from Macklin reagent Co., (Shanghai, China); all of the reagents purchased were analytical grade.

### 2.2. Preparation of Magnetization Modified Cellulose Nanofibrils Composite Materials (MMCNF)

The MMCNFs were prepared by in situ incorporation of Fe_3_O_4_ (as the magnetic response additive). Cellulose nanofibrils (22.22 g hydrogel, real mass of cellulose fiber 1 g) was added to a three-necked flask, followed by adding 80 mL deionized water. The CNFs were fully dispersed by stirring using a homogenizer (Netzsch, Selb, Germany) to give a high-speed shear, and finally the cellulose solution was generated. The flask was then set in a water bath at 75 °C and kept for 30 min. Then, a certain amount of FeCl_3_·6H_2_O (2.4 g) and FeCl_2_·4H_2_O (0.9 g) were added, and the matrix was fully homogenized by vigorous stirring. Ammonia (5.08 g, 25 wt%) was added to adjust the pH value to 9–10 and stirred continued at the fixed temperature for 1 h. The final products were separated using a magnet and then washed with deionized water and ethanol to get rid of potential impurities or water-soluble substances. After that, the purified MMCNFs were immersed in excessive acetone to wash several times, and the MMCNFs were finally dried using freeze-drying equipment.

### 2.3. Preparation of MMCNF/Poly (NIPAM) Semi-IPN Composite Materials (MMCNF-PNA)

**Figure 1:**
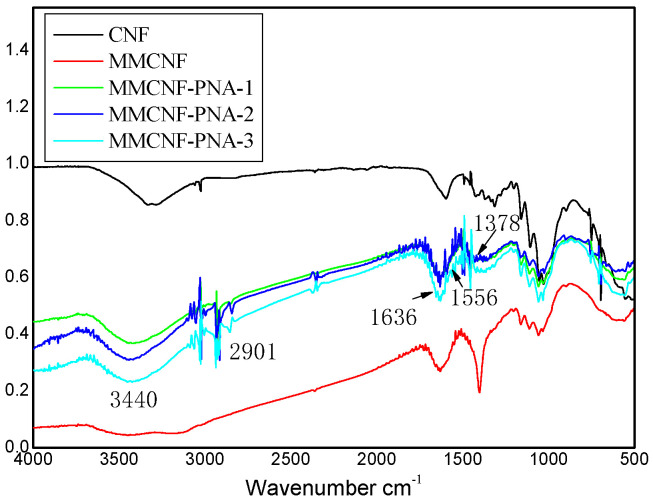
The FTIR spectra of CNF, MMCNF, MMCNF-PNA-1, MMCNF-PNA-2 and MMCNF-PNA-3.

The MMCNF-PNAs were fabricated using an internal crosslinking method of MMCNF via polymerization (free-radical) with a thermal-responsive monomer (NIPAM) in the presence of an initiator and crosslinker (KPS and MBA), respectively [11]. In the process, a certain amount of as-prepared MMCNFs (2 g) was added to a three–necked flask which was heated in a 75 °C water bath, and then deionized water was also added (100 mL). The solution was fully dispersed by stirring, followed by adding the proper amount of NIPAM. The KPS (0.03 g, 0.06 g, 0.12 g), MBA (0.015 g, 0.03 g, 0.06 g), and TEMED (10 μL) were then added to the flasks, and the matrix was heated at 75 °C for 6 h (TEMED was added as an accelerator). NIPAM was added at fixed amount (for different samples, 0.25 g, 0.5 g, and 1.0 g of NIPAM were added). After a 6 h reaction period, the MMCNF-PNA composite materials were soaked in the excess distilled water and ethanol, respectively, and washed three times to remove those potential impurities. The obtained final products were dried using freeze-drying equipment for 24 h under fixed conditions (vacuum and −60 °C). The samples were marked as MMCNF-PNA-1, MMCNF-PNA-2 and MMCNF-PNA-3 in terms of the amount of NIPAM added during the preparation. The recipes are indicated in Table 1 and Figure 1.

### 2.4. Characterization

The characteristic peaks were measured using Fourier transform infrared spectroscopy (FT-IR, Invenio S, Bruker, Billerica, MA, USA). During the test, the samples were ground with a certain amount of KBr and pressed into thin sheets. The resolution was set at 4 cm^−1^. The testing range was set from 4000 cm^−1^ to 500 cm^−1^. A transmission electron microscope (FEI Tecnai G2 F30, Hillsborough, ON, USA) was used for morphological characterization and the component analysis with the Energy spectrum model of Oxford XPLORE. Prior to observation, all the samples were dispersed in water via ultrasound (10 mg in 100 mL). One drop of the solution was dropped on the sample stages and dried, followed by a spray process to cover a thin film of gold before testing. The thermal decomposition of the MMCNF-PNA was measured using a thermogravimetric analyzer (TG) (model Q600, Wilmington, DE, USA) to compare the stability and decomposition peaks. The measurement of about 10 mg of the sample was carried out under N_2_ atmosphere with a temperature range of 25 °C~600 °C and a heating rate of 10 °C/min.

### 2.5. Batch Adsorption

The batch adsorption was carried out using dyes (MB and MV) as the indicator. The influence of shaking time, pH and temperature was systematically investigated as well. First, the stock solution with the concentration of 1000 mg/L was prepared, followed by dilution to obtain tested samples with different concentrations. During the test, 10 mg of the adsorbent was added to 10 mL of dye solution with the concentration of 20 mg/L at a fixed temperature. The matrix was then set on the TS110X30 shaker (Changzhou Jintan Liangyou Instrument Co., Ltd., Changzhou, China), and the tests were carried out with an agitation speed of 150 rpm. After a predetermined period, the concentrations of the dyes were tested using a UV spectrophotometer. The *R* (removal rate, %) and *Q_e_* (adsorption capacity at equilibrium, mg/g) could be obtained based on the results from the test [12]. Three replicates’ measurements were used to make sure of the accuracy of all the experiments.

(1) $$Q_{e}=\left(C_{0}-C_{e}\right)V/m$$

(2) $$R=100(C_{0}-C_{e})/C_{0}$$

where *C*_0_ (mg L^−1^) and *C_e_* (mg L^−1^) represent the initial concentration and the equilibrium concentration; *V* (L) represents the volume; and *m* (g) represents the mass.

### 2.6. Adsorption Mechanism Study (Isotherm Models and Kinetic Models)

The adsorption isotherm models and kinetic models were also used to verify the mechanism of adsorption processes. For the kinetic model, the experiment was designed and carried out with a fixed pH (pH 7) by adding 10 mg of samples in 10 mL of dye solution with an initial concentration of 20 ppm (25 °C and 37 °C); the matrix was set on a shaker at 130 rpm, and the adsorption time was set as 420 min to confirm the adsorption equilibrium. The adsorption capacity at a certain time was measured, and the related kinetic models, including pseudo-second-order, Elovich, and pseudo-first-order models, as well as the corresponding linear relations, were obtained. For the isotherm study, the experiment was carried out with a fixed pH (pH 7) by adding 10 mg samples to 10 mL of dye solution with different initial concentrations (the temperature was set as 25 °C, 37 °C and 65 °C). The matrix was set on a shaker at 130 rpm and the final concentrations of the solution were measured after the sufficient time adsorption process. The isotherm models, including the Freundlich and Langmuir models, were then calculated, and the equations of the kinetics and isotherms are indicated in the Supplementary Materials (See SM) [13].

### 2.7. Desorption and Reusability

After the adsorption process, the dye-loaded adsorbents were taken out and added to 10 mL of HCl solution with a concentration of 1 mol/L at room temperature. The supernatant was then extracted after a certain length of shaking, and the concentration was then measured, followed by the calculation of the desorption efficiency. The desorbed adsorbents were then further washed with distilled water and acetone three times and stored in a sample bag after drying. The recycled adsorbents were then further used to determine the reusability [14].

## 3. Results and Discussion

### 3.1. FTIR

The results of FTIR spectra (MMCNF-PNA-1, 2, 3) are indicated in Figure 1, showing the difference of the CNFs and modified CNFs. In the CNF spectra, peaks at 3340 cm^−1^, 2898 cm^−1^ and 1621 cm^−1^ were observed, corresponding the stretching vibration of O-H bond, the stretching vibration of the C-H bond and the O-H bending, respectively [15]. In addition, the bond at 1065 cm^−1^ related to the stretching vibration of C-O [16]. Meanwhile, the obtained FTIR curves of MMCNF-PNA-1, MMCNF-PNA-2 and MMCNF-PNA-3 revealed the evidence of the chains from PNIPAM with the related bond at 1636 cm^−1^ and 1556 cm^−1^, which corresponded with the N-H stretching and the C=O stretching from amide I, respectively [17]. Furthermore, a peak at 1378 cm^−1^ was also observed, which might be related to the group of PNIPAM (-CH(CH_3_)_2_) [18]. The above results demonstrated that MMCNF-PNA-1, 2 and 3 copolymers were fabricated successfully.

Meanwhile, from Figure 1, the absorbance peak at 3340 cm^−1^, which is related to the -OH group stretching, changed to 3440 cm^−1^ after the copolymerization, which might be due to the reaction of -OH groups. Compared to the result of MMCNF-PNA-1, the peak intensity of MMCNF-PNA-2 and MMCNF-PNA-3 at 1556 cm^−1^ (corresponded to the N-H stretching of NIPAM) increased obviously, showing that a greater quantity of NIPAM monomers was grafted on MMCNFs. The same phenomenon was also indicated by the bond of 1636 cm^−1^ (related to the C=O stretching for amide I).

### 3.2. Scanning Electron Microscope (SEM)

**Figure 2:**
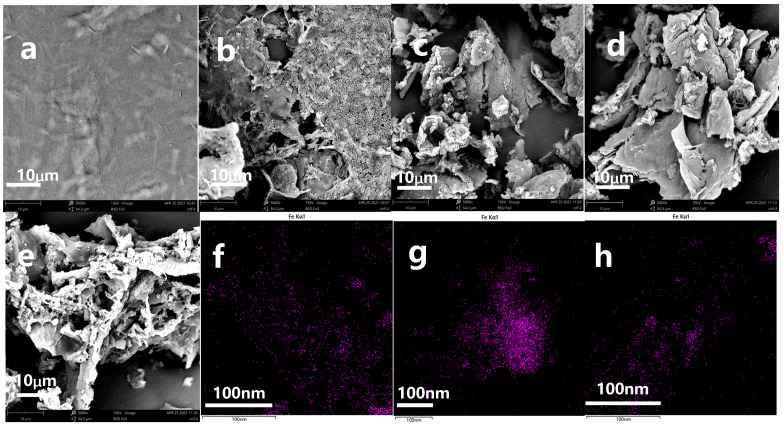
The SEM images of CNF (**a**), MMCNF (**b**), MMCNF-PNA-1 (**c**), MMCNF-PNA-2 (**d**) and MMCNF-PNA-3 (**e**), EDS-Fe element mapping for MMCNF-PNA-1, MMCNF-PNA-2 and MMCNF-PNA-3, the EDS images of Fe element on MMCNF-PNA-1 (**f**), MMCNF-PNA-2 (**g**), MMCNF-PNA-3 (**h**).

The SEM images of CNF, MMCNF, MMCNF-PNA-1, MMCNF-PNA-2, MMCNF-PNA-3 and EDS-Fe element mapping for MMCNF-PNA-1, MMCNF-PNA-2 and MMCNF-PNA-3 are indicated in Figure 2. From Figure 2a, the nano structure of CNF could be observed, in which the film morphology (formed by CNF) was also observed. After magnetic modification (Figure 2b), the individual CNF appeared to be invisible, and the CNF incorporated with Fe_3_O_4_ was clearly indicated. In comparison with the morphology of CNF and MMCNF (see Figure 2a,b), the responsive (semi-IPN) consisting of PNIPAM grafted CNF (Figure 2c–e) appeared to have a more porous structure, which might be due to the polymer having formed a certain number of channels or pores in the composites via grafting [19]. Furthermore, in comparison with the images obtained from MMCNF-PNA-1 (Figure 2c), the more porous structure could be observed on the image of MMCNF-PNA-3 (Figure 2e), implying more polymer chains grafted on MMCNF-PNA-3. The EDS results also indicated that the CNFs incorporated with Fe_3_O_4_ in the matrix (Figure 2f–h).

### 3.3. Thermal Gravimetric Analyzer (TGA)

**Figure 3:**
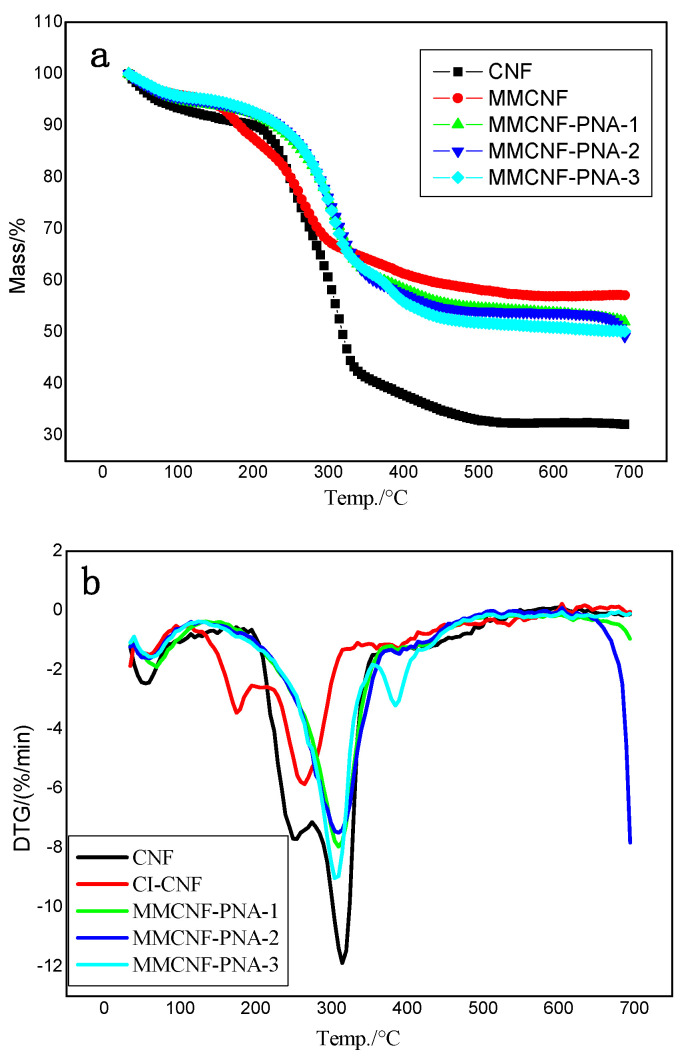
The curves of TG (**a**) and DTG (**b**) (CNF, MMCNF, MMCNF-PNA-1, MMCNF-PNA-2 and MMCNF-PNA-3).

To reveal the thermal properties of CNF, MMCNF, MMCNF-PNA-1, MMCNF-PNA-2 and MMCNF-PNA-3, the TG results are shown in Figure 3a,b. From Figure 3a, the CNF showed the mass loss of 70% with the tested temperature range from 25 °C to 700 °C (Figure 3a). Obviously, the thermal decomposition stages of MMCNF-PNA-3 happened in three parts; the first stage of the thermal decomposition occurred at the temperature range of 50–150 °C, which might be because the water evaporated in the testing samples. The second stage of the thermal decomposition happened at the temperature of 310 °C, which was related to the major thermal decomposition of CNF. The third thermal decomposition stage occurred at 383 °C, corresponding to the degradation of the PNIPAM modified CNF [19,20]. Meanwhile, from the curves shown in Figure 3b, the thermal degradation of the MMCNF-PNA-3 happened at 310 °C with the maximum weight loss observed, showing a 5 °C change lower than that of CNF. Obviously, the PNIPAM grafted CNF showed lower thermal stability than CNFs, which might be due to the grafting destroying the hydrogen bond between the chains of CNFs.

### 3.4. The Contact Time Effects on Adsorption

**Figure 4:**
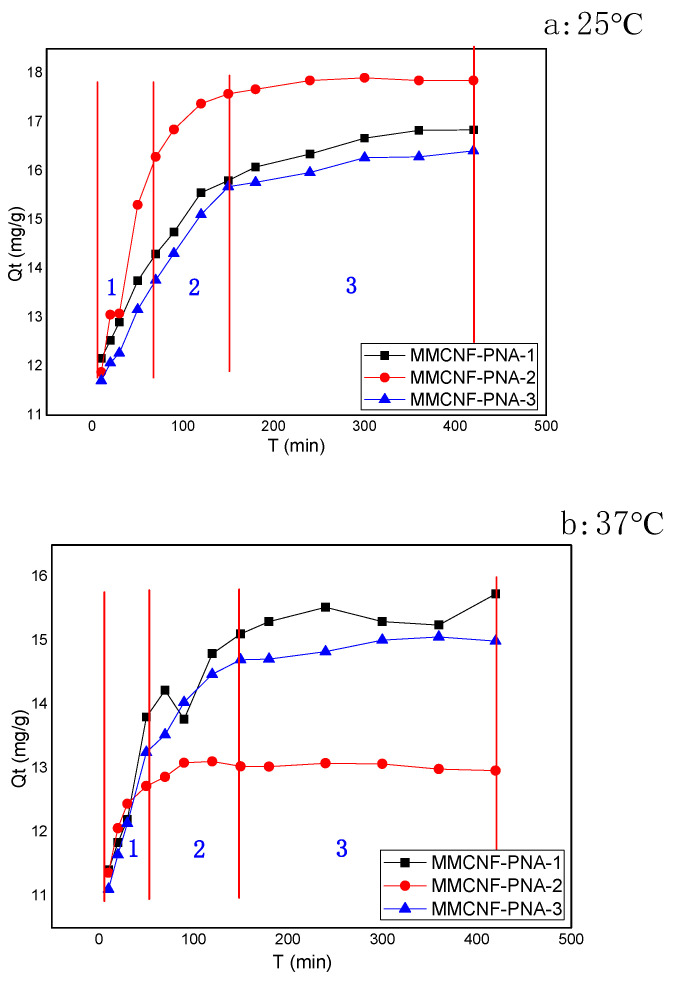
Effect of contact time on adsorption (the area 1, 2, and 3 in the figures are divided to show the different adsorption stages).

The contact time effects on adsorption are indicated in Figure 4. The dye (MB as the indicator) adsorption was investigated under the desired conditions: pH 7; initial concentration of 20 ppm; temperatures fixed at 25 °C and 37 °C; adsorbent dosage at 1000 mg/L. After a 150 min adsorption process, the equilibrium reached for the temperatures was at 25 °C and 37 °C, and the above process were obviously divided into three different events, which are demonstrated in the Figure 4a,b. In the first stage, the time range from 0 to 60 min proved that the adsorption occurred on the external surface. The second stage, between 60 and 150 min, was named as the gradual adsorption process. The third event, which began at 150 min, was named the equilibrium process, where the relatively lower concentration of MB led to a slow intraparticle diffusion process [21]. Interestingly, thermal responsivity was exhibited in Figure 4a,b; the sample MMCNF-PNA-2 had the proper amount of thermal-responsive polymers grafted and thus had the more obvious thermal-responsive behavior, resulting in the decrease of the Qt at 37 °C towards 25 °C. The contraction of NIPAM molecular chains and the changes from hydrophilic to hydrophobic led to a weakened adsorption capacity. Meanwhile, these whole processes were imitated with different kinetic models to verify the adsorption mechanism systematically afterwards.

### 3.5. Adsorption Kinetic Models

**Figure 5:**
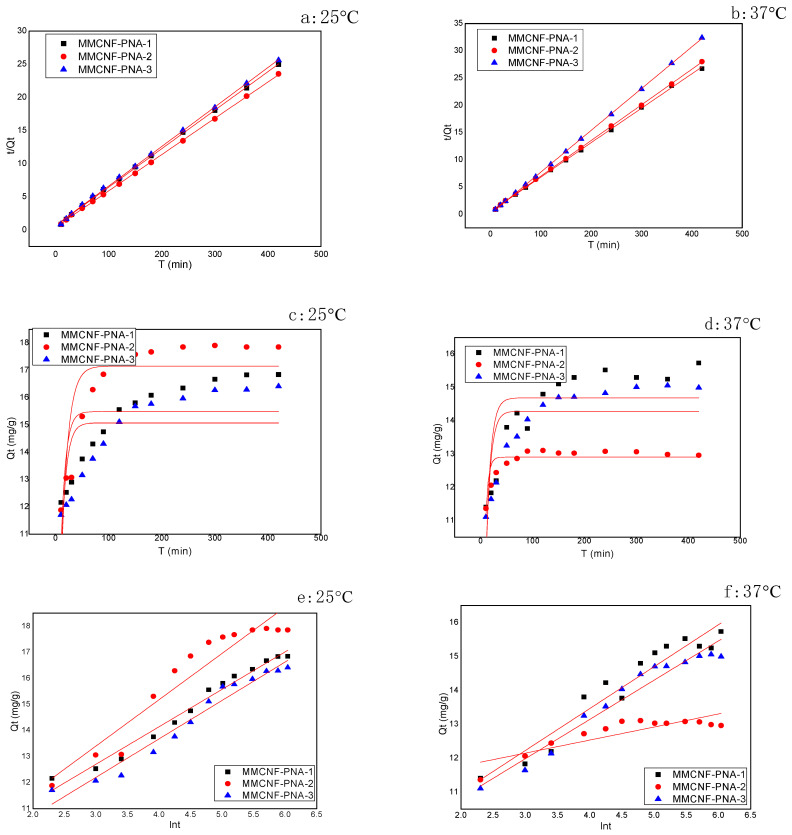
Kinetics of the MB adsorption using MMCNF-PNA-1 (**a**,**b**—pseudo-second-order), MMCNF-PNA-2 (**c**,**d**—pseudo-first-order) and MMCNF-PNA-3 (**e**,**f**—Elovich model).

Adsorption kinetic models were introduced to verify the adsorption mechanism. Herein, three models, pseudo-Second order, pseudo-first order and Elovich, were applied [22]. The pseudo-second order and pseudo-first order models usually can be applied to surface-controlled adsorption processes. Another model, the Elovich model, has been applied on a heterogeneous adsorbent which fits the energetic surface adsorption. The corresponding results of all the models are revealed in Figure 5 and Table 2. The linear relations of the kinetic models are revealed in Figure 5. Obviously, pseudo-second order was the better model to fit the adsorption than the other two models (pseudo-first order model and Elovich model), showing that the adsorption mechanism was chemical adsorption onto MMCNF-PNA-1, MMCNF-PNA-2 and MMCNF-PNA-3. In comparison with the k_2_ values of MMCNF-PNA-1 and MMCNF-PNA-2 at a temperature of 25 °C, the k_2_ value at 37 °C was higher, showing a faster dye uptake ability, which might be because of the more intense movement of molecular chains at high temperatures.

### 3.6. Adsorption Isotherms

**Figure 6:**
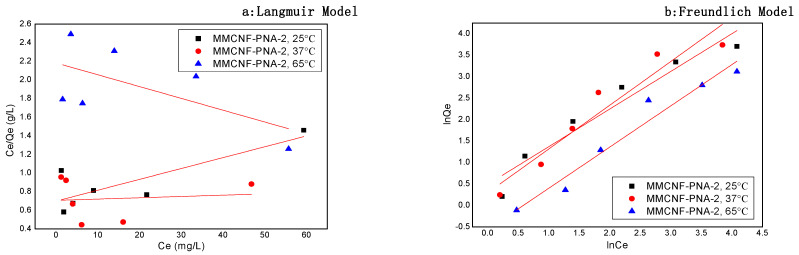
The adsorption isotherms for the adsorption of MB on MMCNF-PNA-1, MMCNF-PNA-2 and MMCNF-PNA-3: (**a**) Langmuir model and (**b**) Freundlich model.

The adsorption isotherms were studied using the Langmuir model and Freundlich model, and the related results are revealed in Figure 6 and Table 3. As revealed in Figure 6, the Freundlich plot of lnQ_e_ versus lnCe exhibited the higher R^2^ (correlation coefficients), showing a better linear relationship, especially at high temperature (Figure 6b and Table 3, 65 °C). Conversely, the Langmuir isotherm model revealed the lower R^2^, showing a poorer fit at all temperatures. The Freundlich model fitted well to the dye adsorption on MMCNF-PNA-2, showing that the adsorption process was attributed to the heterogeneous adsorptive energies. Furthermore, the MMCNF-PNA-2 showed a higher R^2^ while using the Langmuir isotherm model to fit the data at low temperatures, indicating the fact that the dye adsorption on MMCNF-PNA-2 was more like a monolayer adsorption below the responsive temperature of the adsorbents.

Generally, the K_F_ values indicate the adsorption capability. As indicated in Table 3, the decrease of the temperature shows the increase of the K_F_ value, resulting in the higher adsorption capacity towards dye, which might be due to the more porous structures exhibited by adsorbents at low temperatures. These pores are beneficial for adsorption. In general, the n values from Freundlich model (empirical coefficient) means different results (2–10 represents good adsorption capability, 1–2 represents moderately difficult adsorption capability, and less than 1 represents poor adsorption capability). From the values shown in Table 3, n was around 1 for the all samples, indicating a moderately difficult adsorption capability [23]. Table 4 compared the adsorption capacities between the MMCNF-PNA-2 and the other similar reported cellulose-based adsorbents; the results indicated that the adsorbent prepared in this work had a relatively high adsorption capacity towards cationic dyes, such as MB.

### 3.7. The Desorption and Recycling of Adsorbents

**Figure 7:**
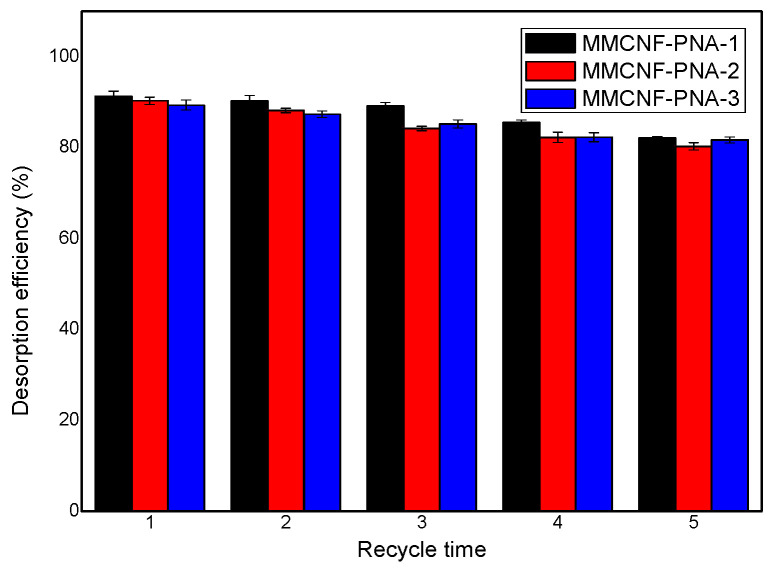
Desorption behavior of MB on adsorbents.

Desorption and reusability were studied and the related results are shown in Figure 7. As indicated in Figure 7, the desorption efficiency for all the samples were obtained to be 82.12%, 80.26% and 81.69% of MB for MMCNF-PNA-1, MMCNF-PNA-2 and MMCNF-PNA-3 at the fifth recycle, respectively. The recovered adsorbents were applied to adsorb MB again (10 mg in 10 mL of 20 ppm MB solution, 2 h adsorption time) and the removal efficiency (R%) was tested. The R% for MMCNF-PNA-1, MMCNF-PNA-2 and MMCNF-PNA-3 were 75.21%, 73.42% and 81.23%, respectively, showing an excellent reusability. To sum up, the adsorbent exhibits an excellent ability for the desorption and reusability of cationic dyes.

### 3.8. The Study of Thermal Responsive Behavior of the Adsorbents

**Figure 8:**
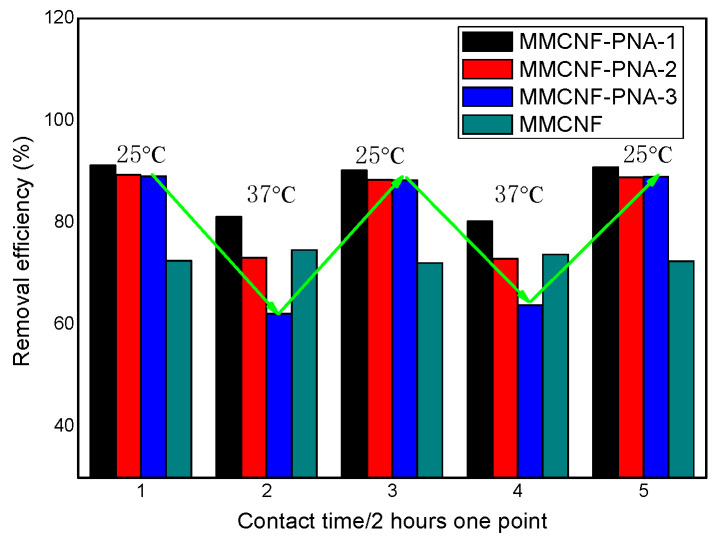
The thermal-responsive behavior of the adsorbents.

The thermal-responsive behavior of the adsorbents was studied and simulated to better describe the responsiveness of the adsorbents compared with the conventional adsorbents (MMCNF). The experiments were carried out by adding 10 mg of each adsorbent to 10 mL of the dye solution at a concentration of 10 ppm. The matrix was set on the shaker mentioned above at 25 °C and kept for 2 h to reach equilibrium. The temperature was fixed to 37 °C and kept for another 2 h to reach equilibrium. The above processes were repeated, and the removal efficiency (R%) of the dye was calculated. The corresponding results are indicated in Figure 8. As indicated in Figure 8, the adsorption equilibrium was reached at around 2 h into the adsorption period at room temperature. The removal efficiency was 91.2%, 89.3%, 89.1% and 72.5% for MMCNF-PNA-1, MMCNF-PNA-2, MMCNF-PNA-3 and MMCNF, respectively. After another adsorption process at 37 °C, the removal efficiency changed to 81.1%, 73.1%, 62.1% and 71.6%, respectively. The R% of MMCNF did not change, and the R% of MMVNF-PNA decreased obviously (especially for MMCNF-PNA-3, from 89.1% to 62.1%) and the R% returned to the initial position while the temperature changed back to 25 °C, showing that the thermal-responsive property would affect the adsorption of the adsorbents, resulting in the possibility to control the dyes adsorption by only changing the adsorption conditions (such as temperature).

## 4. Conclusions

Three responsive adsorbents consisting of nanocellulose/thermal-responsive polymer were fabricated successfully using the common free radical polymerization. The as-prepared CNF-based adsorbents exhibited porous structure and relatively high thermal responsibility and excellent dyes uptake ability. The controlled adsorption and desorption process towards dyes was proved, which might be due to the stimuli responsiveness. Different isotherm and kinetic models were applied to investigate the adsorption mechanism. The corresponding results indicated that adsorption kinetic and isotherm followed pseudo second order and Freundlich model, suggesting that the dyes adsorption occurs by heterogeneous adsorptive energies on the as-prepared semi-IPN. Overall, the novel adsorbents reported herein provides a new strategy to remove water contamination using those novel cellulose-based environmentally friendly composites.

## Figures and Tables

**Table 1:** The mass of MMCNF, AA, NIPAM, MBA and KPS used.

Sample	MMCNF (g)	NIPAM (g)	KPS (g)	MBA (g)
MMCNF-PNA-1	2	0.25	0.03	0.015
MMCNF-PNA-2	2	0.5	0.06	0.03
MMCNF-PNA-3	2	1.0	0.12	0.06

**Table 2:** The R^2^ (correlation coefficients) and the constants for kinetic models.

Sample	*q*_e,exp._ (mg g^−1^)	Pseudo-First Order			Pseudo-Second Order			Elovich Model		
		*a*	*b*	R^2^	*q*_e2,cal_ (mg g^−1^)	*k*_2_ (mg g^−1^ h^−1^)	R^2^	*α*	*β*	R^2^
MMCNF-PNA-1 (25)	17.72	15.48	0.11	0.365	17.24	0.005	0.999	167.25	0.563	0.902
MMCNF-PNA-2 (25)	17.24	17.15	0.08	0.643	18.51	0.007	0.999	288.76	0.678	0.959
MMCNF-PNA-3 (25)	16.96	15.07	0.11	0.356	16.95	0.005	0.999	496.41	0.696	0.976
MMCNF-PNA-1 (37)	15.65	14.68	0.11	0.480	15.38	0.009	0.999	1217.9	0.809	0.924
MMCNF-PNA-2 (37)	14.32	12.91	0.20	0.727	12.99	0.116	0.999	3888.3	0.722	0.718
MMCNF-PNA-3 (37)	15.63	14.28	0.12	0.517	15.87	0.008	0.999	1819.3	0.864	0.948

**Table 3:** Isotherm model constants and correlation coefficients (R^2^).

Samples	T (°C)	Freundlich Constants			Langmuir Constants		
		*K_F_*	*n*	R^2^	*Q_max_*	*K_L_*	R^2^
MMCNF-PNA-2	25	1.634	1.140	0.919	8.57	0.017	0.611
	37	1.342	0.980	0.905	714.28	0.002	0.118
	65	0.571	1.042	0.947	78.8	0.006	0.222

**Table 4:** The adsorption capacities of dyes onto MMCNF-PNA-2 and other adsorbents.

Materials Reported	Adsorption Capacity (mg/g)
Modified cellulose adsorbents [24]	CI Reactive Blue 21 dye: 200
Cellulose derivative [25]	MB: 135
MMCNF-PNA-2 (this work)	MB: 192
Cationized cellulose [26]	Methyl orange (MO): 76.9
Porous microcrystalline cellulose [27]	MB: 82

## Data Availability

Data are contained within the article and Supplementary Materials.

## Supplementary Materials

The following supporting information can be downloaded at: https://www.mdpi.com/article/10.3390/polym16111446/s1, Supplementary data included in the SD section, the formulas of adsorption kinetics and isotherms. References [28,29,30,31] are cited in the Supplementary Materials.
