# Supplementary material for: Preparation and Application of Responsive Nanocellulose Composites
Source: Polymers (Basel). 2024 May 21;16(11):1446. doi: 10.3390/polym16111446 (PMC11174569; doi:10.3390/polym16111446)
Supplement: Supplementary file 1 [file polymers-16-01446-s001.zip › polymers-2959769-supplementary.pdf]

Supplementary Materials for

Preparation and application of responsive nanocellulose composites

Yanhui Zhou, [1] Zhang Lu, [2] and Yuan Li [2,*]

[1] *School of Applied Foreign Languages, Guangdong Industry Polytechnic, Guangzhou, Guangdong 51300, P.R. China*

[2] *Guangdong Biomaterials Engineering Technology Research Center, Institute of Biological and Medical Engineering, Guangdong Academy of Sciences, Guangzhou, Guangdong 510316, P.R. China*

* Correspondence to: yli7@unb.ca (Y. L.)

Adsorption kinetics

The adsorption kinetic models can well predict the adsorption mechanism. In this work, three kinetic models (including the pseudo-first-order, the pseudo-second-order [28], Elovich kinetic models [29]) were used to fit the adsorption data. The models are presented as follows:

$$ln(q_e - q) = lnq_e - k_1 t \qquad (1)$$

$$t/q_t = 1/(k_2 q_e^2) + t/q_e \qquad (2)$$

$$q_t = (\ln \alpha\beta)/\beta + (\ln t)/\beta \qquad (3)$$

Where q_t and q_e are the adsorption capacity at time t and at equilibrium (mg g^{-1}), respectively. K_1 is the pseudo-first-order equilibrium rate constant (h^{-1}), and K_2 is the pseudo-first-order equilibrium rate constant (g mg^{-1} h^{-1}). α and β are the initial adsorption rate at zero coverage and the desorption constant related to the extent of surface coverage and activation energy for chemisorption in the Elovich model.

Adsorption isotherms

The isotherm tests were carried out at the fixed conditions by agitating 10 mg of MMCNF-PNA-2 in 10 mL of dye solution at different initial concentrations on a shaker at 130 rpm under controlled temperature of 25, 37 and 65°C, respectively. The concentrations of dye solution were measured after 3 hours' adsorption. Two isotherm models, including Langmuir and Freundlich models were used to calculate the adsorption results. The linear equations are as follows [30]:

The Langmuir model:

$$C_e/Q_e = 1/(K_L Q_{max}) + C_e/Q_{max} \qquad (4)$$

where Q_{max} (mg g^{-1}) is the maximum adsorption capacity and K_L (L mg^{-1}) is the Langmuir constant. C_e (mg L^{-1}) and Q_e (mg g^{-1}) are the concentration and adsorption capacity at the equilibrium, respectively.

The Freundlich model [31]:

$$ln\, Q_e = ln\, K_f + (ln\, C_e)/n \qquad (5)$$

Where Q_e and C_e are noted previously. K_f is roughly an indicator of adsorption capacity and n is adsorption intensity.

The results of AFM (Phase images)

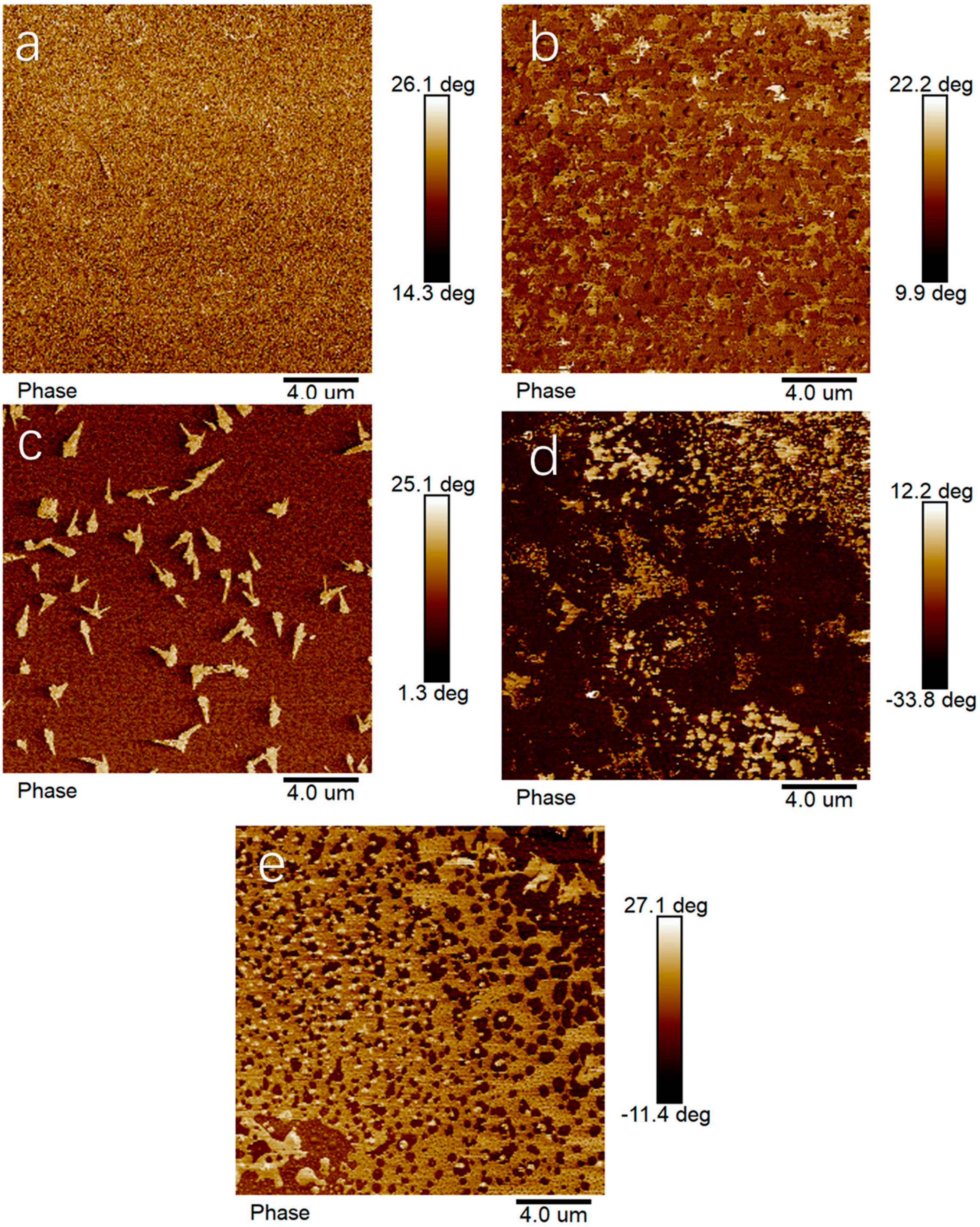

Figure S1. The results of AFM (Phase images: a. CNF; b. MMCNF; c. MMCNF-PNA-1; d. MMCNF-PNA-2; e. MMCNF-PNA-3)

The effects of temperature on adsorption

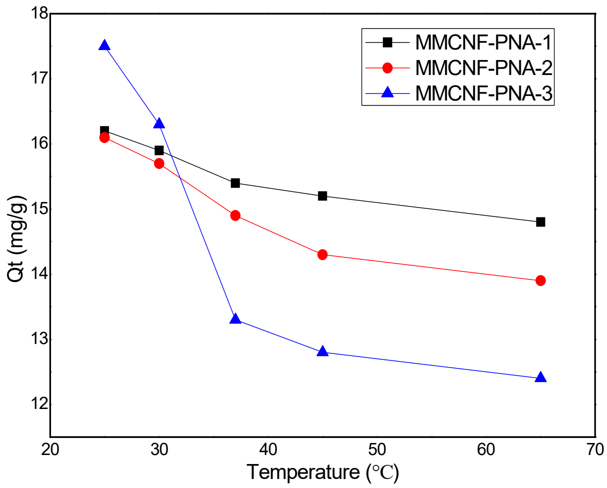

Figure S2. The effects of temperature on adsorption (MB with the initial concentration of 20 ppm; contact time of 2 hours; volume: 10 mL; adsorbent dosage: 10 mg and pH 7)

The effects of temperature and the thermal responsibility were exhibited in Figure S2, the sample MMCNF-PNA-2 and MMCNF-PNA-3 had more amount of thermal responsive polymers grafted, thus had the more obvious thermal responsive behavior, resulting in the decrease of the Qt at 37 °C, 45 °C, 65 °C towards 25 °C. As indicated, the contraction of NIPAM molecular chains and the changes from hydrophilic to hydrophobic lead to the weakened adsorption capacity.

The effects of pH on adsorption

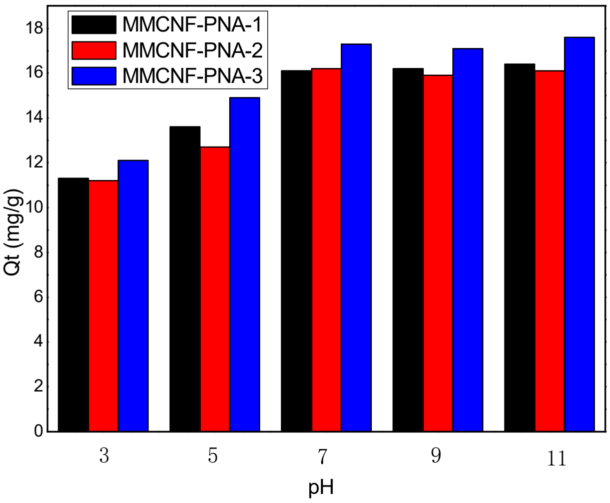

Figure S3. The effects of pH on adsorption (MB with the initial concentration of 20 ppm; contact time of 2 hours; volume: 10 mL; adsorbent dosage: 10 mg and 25 ℃)

The effects of pH were studied and the corresponded results were indicated in Figure S3. The adsorption capacity of the absorbents was relatively lower for pH 3 and 5 than that of pH 7, 9, and 11, due to the fact that the acidic condition is not favorable for the adsorption of cationic dyes in the solution. During the process, the hydrogen ion (H+) will compete with the dye molecular, resulting in the less adsorption capacity obtained. In addition, the alkali condition almost has no impact on the adsorption capacity of the adsorbent, there are no obvious difference when the pH is 7, 9, and 11.

[28] Lin, Q.; Gao, M.; Chang, J.; Ma, H. Adsorption properties of crosslinking carboxymethyl cellulose grafting dimethyldiallylammonium chloride for cationic and anionic dyes. *Carbohydr. Polym.* **2016**, *151*, 283-294.

[29] Zhou, H.; Gao, B.; Zhou, Y.; Qiao, H.; Gao, W.; Qu, H.; Liu, S.; Zhang, Q.; Liu, X. Facile preparation of 3D GO/CNCs composite with adsorption performance towards [BMIM][Cl] from aqueous solution. *J. Hazard. Mater.* **2017**, *337*, 27-33.

[30] Ling, L.L.; Liu, W.J.; Zhang, S.; Jiang, H. Magnesium Oxide Embedded Nitrogen Self-Doped Biochar Composites: Fast and High-Efficiency Adsorption of Heavy Metals in an Aqueous Solution. *Environ. Sci. Technol.* **2017**, *51*, 10081-10089.

[31] Zhao, F.; Repo, E.; Yin, D.; Meng, Y.; Jafari, S.; Sillanpää, M. EDTA-Cross-Linked β-Cyclodextrin: An Environmentally Friendly Bifunctional Adsorbent for Simultaneous Adsorption of Metals and Cationic Dyes. *Environ. Sci. Technol.* **2017**, *51*, 10570-10580.
